# Association between the Occurrence of Primary Hypothyroidism and the Exposure of the Population Near to Industrial Pollutants in São Paulo State, Brazil

**DOI:** 10.3390/ijerph16183464

**Published:** 2019-09-18

**Authors:** Maria Angela Zaccarelli-Marino, Rudá Alessi, Thalles Zaccarelli Balderi, Marco Antonio Garcia Martins

**Affiliations:** 1 Internal Medicine Department, Endocrinology Service, ABC Medical School, Foundation, Santo André, SP 09060-870, Brazil; 2 Internal Medicine Department, ABC Medical School Foundation, Santo André, SP 09060-870, Brazil; ruda.alessi@gmail.com; 3 Internal Medicine Service, Santa Paula Hospital, São Paulo, SP 04556-100, Brazil; thalles@institutoneurologico.com.br; 4 Department of Environmental Health, Harvard T.H. Chan School of Public Health, Boston, MA 02115, USA; mmartins@hsph.harvard.edu

**Keywords:** primary hypothyroidism, petrochemical complex, industrial pollutants, São Paulo state, Brazil

## Abstract

Background: Environmental agents interfere with thyroid function at multiple levels. This study was to investigate the association between pollutant concentrations and the primary hypothyroidism (PH) occurrence odds in residents living in the Capuava Petrochemical Complex (CPC) influence area. Methods: This area was evaluated with the combination of the AERMOD dispersion model with the Weather Research Forecast (WRF) meteorological model (2016). The concentration of atmospheric pollutants were analyzed in 2017 using meteorological data on the period from 2005 to 2009, correlating this data with the research done in 2003 to 2005. A home-based questionnaire was applied to evaluate 2004 residents, of both sexes, aged from 8 to 72 years, based on their proximity to the industrial areas; were select residents with PH. Results: Volatile organic compounds (VOCs), carbon monoxide (CO), and nitrogen dioxide (NO_2_) concentrations presented the highest correlations between the PH odds and pollutant concentrations. Conclusion: Air pollution associated with the presence of the CPC is an important environmental factor contributing to the development of PH in the nearby population. As the first study showing this association in Brazil, research should be continued to better understand the mechanisms and to find ways to compensate for or remedy to avoid health impacts in future populations.

## 1. Introduction

Environmental agents interfere with thyroid function at multiple levels, including thyroid hormone synthesis, thyroid hormone metabolism and excretion, and thyroid hormone action [1,2,3,4]. 

Primary hypothyroidism (PH) is the most common thyroid pathology [5] and its frequency has been increased in recent years [6,7]. 

Several large population-based screening studies have reported the prevalence of overt hypothyroidism to be around 1 to 3% [5,8]. In community surveys, the prevalence of overt hypothyroidism varies among 0.1 and 2% [6,9,10]. The Vanderpump and Tunbridge study [11] the spontaneous hypothyroidism prevalence is between 1% and 2%, it is more common in older women, and 10 times more common in women than in men. 

According to Sgarbi et al. the incidence of hypothyroidism among 1110 individuals (≥30 years old) from a Japanese-Brazilian population of Bauru, was 11.1% in females and 8.7% in males [12].

Autoimmune thyroid diseases prevalence is about 5% [6,13]. The study performed in Whickham [6] demonstrates that patients who present positive antithyroid antibodies are highly likely to develop hypothyroidism.

Environmental factors such as atmospheric pollutants are presently being studied as an important cause of thyroid autoimmune disease (TAD) [14]; it is the most common organ-specific autoimmune disorder, affecting 2% to 5% of the population in Western countries [15], especially adult women and the elderly [7,16].

According to Rádiková et al. [14], the prevalence of A-TPO is significantly higher in both men and women in areas that are polluted with nitrates and organochlorines.

A large long-term study in Slovakia measured polychlorinated biphenyls (PCB) exposure in adults’ individuals and found an association with higher exposure and an increase in thyroid volume, serum thyroid stimulating hormone (TSH), and thyroid autoantibodies, especially in women [16]. Exposure to polyhalogenated biphenyls and polyhalogenated biphenyl oxides in male factory workers was associated with an increased incidence of antimicrosomal thyroid antibodies and hypothyroidism [17]. Organic pollutants, such as polyaromatic hydrocarbons, have also been associated with goiter and thyroid disease [18].

Evidence suggests that, in most industrialized countries, autoimmune disorders—including chronic lymphocytic thyroiditis—are increasing [19].

São Paulo State (SP) is the most populous and industrialized state in Brazil with about 45 million inhabitants and 7012 industries [20]. In our previous studies [21,22], which were conducted on a densely populated area of SP, surrounding the Capuava Petrochemical Complex (CPC), we reported overt primary hypothyroidism [22] and the increasing of chronic autoimmune thyroiditis (CAT) incidence over the years [21]. Petroleum processing can emit different organic compounds that can threaten human health [23].

As few data are available on the role of pollution from petrochemical complexes, and the existence of CPC in Santo André, in Sao Paulo metropolitan region, that are producing polyethylene and polypropylene from naphtha distillation, as well as various intermediate substances used as raw material for manufacturing other composites or for the market [24], we investigated the possible role of environmental pollution in PH.

The objective of this study was to investigate the association between pollutant concentrations: nitrogen dioxide (NO_2_), carbon monoxide (CO), particulate matter (PM_10_), sulfur dioxide (SO_2_), and volatile organic compounds (VOCs) in the atmosphere and the primary hypothyroidism (PH) occurrence odds in residents living in the Capuava Petrochemical Complex (CPC) influence area.

## 2. Methods

### 2.1. Atmospheric Pollutants

The concentration of atmospheric pollutants were analyzed in 2017 by numerical simulations with the AERMOD model, using meteorological data on the period from 2005 to 2009, physical characteristics of the environment (topography and type of land use), and the pollutant sources (information from the physical characteristics of the sources and theirs emissions), correlating this data with the research done in 2003 to 2005.

**Figure 1:**
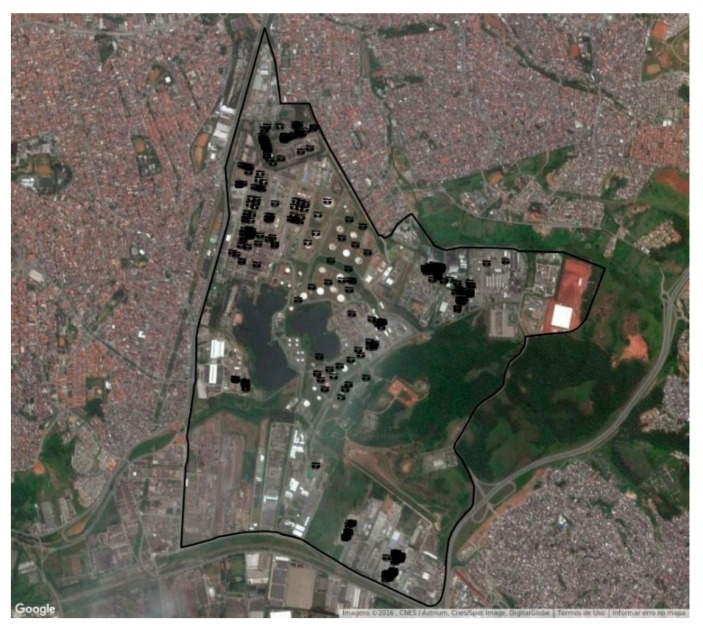
Satellite image showing the CPC area and evaporative emission sources in black.

The AERMOD is a dispersion model developed by the American Meteorological Society (AMS) and the U.S. Environmental Protection Agency (EPA) [25] and made available for public use. The area of influence of the CPC atmospheric emissions was evaluated with the combination of the AERMOD dispersion model with the Weather Research Forecast (WRF) meteorological model (American Meteorological Society/Environmental Protection Agency Regulatory Model 2016) [26]. According to recommendations from the Environmental Protection Agency from USA (EPA) [25], the dispersion model has to be applied for 5 years of meteorological data. This was accomplished by the fields provided by the WRF model; with the combination of the meteorological fields with the emissions of the pollutants, the concentrations isopletes for the regulated pollutants were calculated for the receptor grid [26]. The map of the concentrations for each pollutant was used to evaluate the hot spots where population is more exposed. The only documentation on the building downwash algorithm in AERMOD (American Meteorological Society/U.S. Environmental Protection Agency Regulatory Model), referred to as PRIME (Plume Rise Model Enhancements), recent field, and wind tunnel studies have shown that AERMOD can overpredict concentrations by factors of 2–8 for certain building configurations. While a wind tunnel equivalent building dimension study (EBD) can be conducted to approximately correct the overprediction bias, past field and wind tunnel studies indicate that there are notable flaws in the PRIME building downwash theory. Although AERMOD/PRIME may provide accurate and unbiased estimates (within a factor of 2) for some building configurations, a major review and update is needed so that accurate estimates can be obtained for other building configurations where significant overpredictions or underpredictions are common due to downwash effects [27]. Two categories of pollutants were analyzed: 1) Volatile organic compounds (VOCs) which have its main source the evaporation and leakage of the storage tanks; and 2) Pollutants controlled by the São Paulo State legislation: NO_2_, CO, PM_10_, and SO_2_ those are emitted from chimney after processing (Figure 1). Due to the impact on health, the dispersion curves of VOCs were also analyzed, even though the Brazilian legislation does not regulate VOC concentrations.

### 2.2. Study Design and Data Collection

This is a cohort study. We have evaluated residents living in the Capuava Petrochemical Complex (CPC) influence area during the period of July 2003 through June 2005. The research population was defined starting with 2004 residents after the explanation about this study. Previously to data collected, free consent was signed by the father, mother, or responsible person and authorization to this realization was obtained from the residents.

The data collected consisted in obtaining information on the residents, and a home-based questionnaire was applied to evaluated residents from 2004 of both sexes, aged 8–72 years old, divided into two groups (A and B) based on their proximity to the industrial areas. The survey was formulated and applied by the authors of this study.

The questions asked on the survey were about: Identification-name (initials), age (years), sex (male, female), address and time at local residency, profession and education (adults), education (children and adolescents) and social and economic situation (adults); medical history—thyropathies, hypothyroidism (previous diagnosis of PH realized by doctor and prescription); hyperthyroidism, thyroid surgery, radioactive iodine, use of thyroid hormone (TH), age at initial treatment with TH (after eight years of age), thyroid nodules, malignant thyroid nodules, previous treatment with radioiodine or radiotherapy of the chest and neck and craniotomies; medicines—amiodarone, lithium carbonate, iodine, interferon—because these medications could trigger PH; thyroid-specific medications, use of TH, thioanamides (methimazole and propylthiouracil) and in children and adolescents, learning, and growth (good, average, or poor).

Group A: comprised 1002 residents, females and males, living in the surroundings of the CPC. This industrial area was named Region 1, and is occupied by 14 industries producing polyethylene and polypropylene from naphtha distillation and various intermediary substances that are used as raw materials for manufacturing other products. The area is located on the boundaries of Santo André, Mauá and São Paulo, State of São Paulo (SP), Brazil (0.5 km, 1.0 km, and 2.0 km away from CPC, respectively).

Group B: comprised 1002 residents, males and females, living in the surroundings of different industrial area, mainly steel industries with no petroleum byproducts manufactured. The area is located on the boundaries of Santo André, São Bernardo do Campo, and São Caetano do Sul, SP, Brazil (7.5 km, 8.0 km, and 8.5 km away from Region 1, respectively). This industrial area was named Region 2 and was treated as the control group.

Regions 1 and 2 were each divided into three districts; 334 residents were evaluated in each district.

Each resident had lived in either Region, 1 or 2, for more than 10 years in the same home, and the controls were matched for similar social and economic situations. The socio-economic conditions were evaluated through the questionnaire, and the residents were selected when they had similar salaries and social habits (these residents do not have the economic needs to move far from the polluted areas, according to the subjects themselves).

The residents were considered adults if they were over or equal to 18 years of age, and children and adolescents if they were under the age of 18. When the residents were children or adolescents, the questions were presented to the parents or responsible persons.

The visits occurred once in each house, and to guarantee the maximal participation we included weekends.

During the study time (2003–2005), there was no pre-selection of residents for Regions 1 and 2, and only spontaneous answers were considered.

The inclusion criteria for the analysis of data included ages between 8–72 years, each resident had lived in either Region 1 or 2 for more than 10 years in the same home, and only residents presenting with PH without other thyroid pathologies and affirmative answers for the use of thyroid hormone (TH).

Residents who were taking medications that could trigger PH—including amiodarone, lithium carbonate, iodine, interferon, and thionamides (methimazole and propylthiouracil)—were excluded together with workers of the CPC that lived in the Region 1 and 2.

Residents with hyperthyroidism, malignant nodules or previous thyroid treatment with radioiodine or radiotherapy of the chest and neck and patients from other regions were also excluded. In addition, patients who submitted to thyroidectomies, use of radioactive iodine, radiotherapy, and craniotomies and residents who began TH within the first eight years of life to eliminate congenital primary hypothyroidism (congenital PH) or central hypothyroidism (pituitary or hypothalamic) disorders were also excluded.

Six areas were delimited using the streets where the questionnaires were performed: on Region 1—Location (L): L1—Santo André-Capuava Park, L2—Mauá-Sílvia Maria and Sonia Maria Gardens and L3—São Paulo-São Rafael Park; on Region 2—Location: L4—Santo André-Prince of Walles Villa, L5—São Bernardo do Campo-Vivaldi Villa, and L6—São Caetano do Sul-Santa Maria District.

The average concentrations of each pollutant, for each of the six districts considered were calculated using the simulated plumes.

### 2.3. Ethical Statement

This research was approved by the Committee of Ethics in Research of the ABC School of Medicine, SP, Brazil and registered under number 087/2002.

The objectives and methods of this study were clearly stated to all residents or to parents in the case of children. All residents agreed to participate in this study.

## 3. Statistical Analysis

The odds of PH in each district were computed for each combination of sex and age and compared by means of Wald tests with Bonferroni correction for multiple comparisons. The association between pollutant concentrations and the odds of PH was evaluated by Spearman coefficients of correlation for each combination of the categories of sex and age. A polynomial regression having the logarithm of the odds of PH as response and sex, age, and pollutant concentration up to the third degree along with their interactions was fitted to the pollutant which exhibited a consistent trend of increase in concentration with increasing distance from the CPC. The adopted model was selected via a backward elimination procedure and goodness of fit was evaluated via residual analysis.

## 4. Results

Table 1 shows the frequencies of residents with PH in each combination of sex and age categories.

Multiple comparisons indicated that the odds of PH corresponding to the districts closer to the CPC are similar, and different from those corresponding to the distant districts that are similar among themselves.

Based on the dispersion model, it was possible to estimate where the maximum values of each pollutant concentration were found.

Average concentrations of NO_2_, CO, PM_10_, SO_2_, and VOCs obtained from the simulated plumes for the six districts are presented in Table 2. The three Districts closer to the CPC showed higher average concentrations for all pollutants compared to the districts located further. Therefore, only NO_2_, CO, and VOCs concentrations presented a tendency to decrease with distance from CPC.

**Figure 2:**
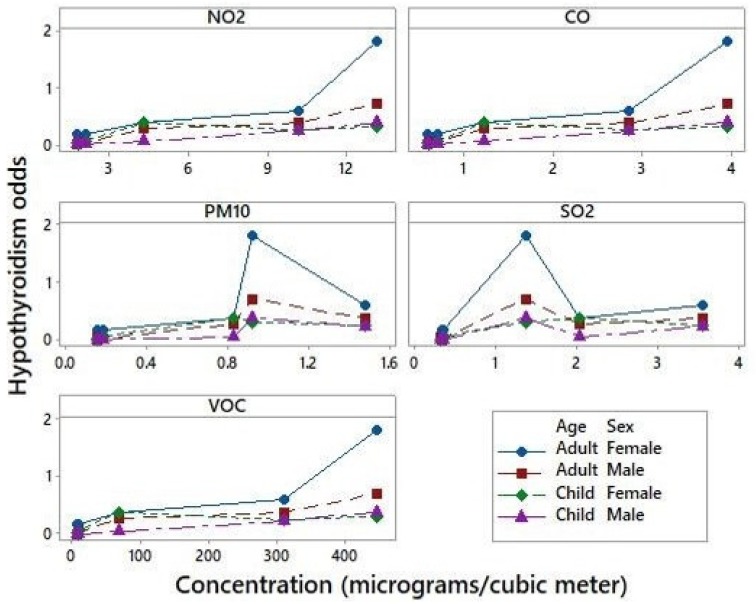
Scatterplots showing the Hypothyroidism odds related to NO2, CO, PM10, SO2, and VOC concentrations.

The associations between odds of PH and pollutants concentrations are displayed in Figure 2. The odds for PH showed a clear trend to rise when NO_2_, CO, and VOCs concentrations increased, and this pattern is not observed for PM_10_ and SO_2_.

The Spearman correlation coefficients between the odds of PH and pollutants concentrations are presented in Table 3. NO_2_, CO, and VOCs concentrations showed the highest correlations. They are also highly correlated among themselves (Pearson correlation coefficient = 0.99).

**Figure 3:**
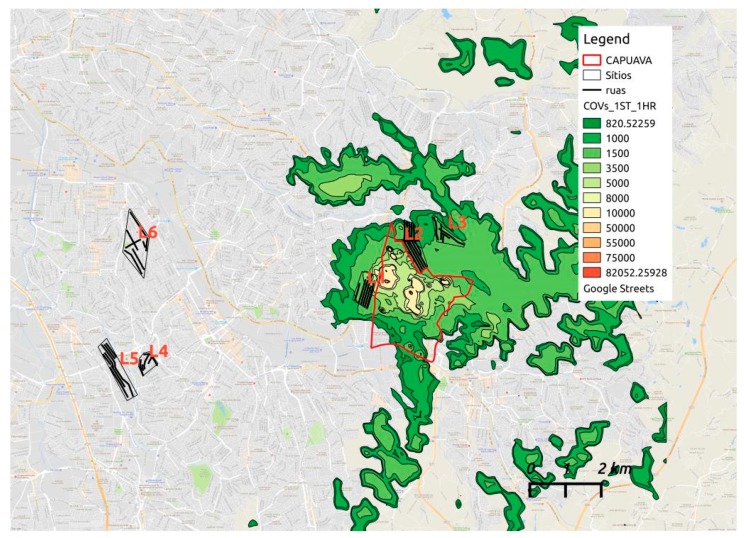
VOC maximum per hour average concentrations for the period between January of 2005 and December of 2009 using AERMOD simulation. Black lines are streets where epidemiologic studies were realized: L1—Capuava Park–Santo André, SP; L2—Sílvia Maria and Sonia Maria Gardens–Mauá, SP; L3—São Rafael Park–São Paulo, SP; L4—Prince of Walles Villa–Santo André–, SP; L5—Vivaldi Villa–São Bernardo do Campo, SP; L6—Santa Maria District–São Caetano do Sul, SP.

Figure 3 shows the highest values of VOCs localized in the proximities west and north of CPC, and the values higher are within the limited area of the CPC, where the questionnaires were performed with a significant frequency of PH (Table 1).

## 5. Discussion

According to our results, the associations between odds of PH and pollutants concentrations are displayed in Figure 2. The odds for PH showed a clear trend to rise when NO_2_, CO, and VOCs concentrations increased, and this pattern is not observed for PM_10_ and SO_2_.

Our findings showed the highest values of VOCs localized in the proximities west and north of CPC (Figure 3), but the values that are higher than the pattern of air quality are within the limited area of the CPC, where the questionnaires were performed with a significant frequency of PH (Table 1).

There are numerous emission sources in the petroleum refining and petrochemical industries [28].

According to Uzma et al. [29], there is increasing concern regarding the possible linkage between chronic exposure to both solvents (benzene) and air pollutants (carbon monoxide) with thyroid functioning.

Another study, conducted around a major petroleum refinery in Greece, also concluded that aromatic hydrocarbons are a major source of VOCs, saturated hydrocarbons are also prevalent [30] which originated from the evaporation of oil products.

Polycyclic aromatic hydrocarbons (PAHs) have two or more fused aromatic rings. They are classified as a group of organic pollutants [31] that arise from anthropogenic activities, such as oil shipping and refineries. These compounds have adverse effects on development and function of the thyroid gland in mammals [31]; however, there are few studies on the effects of PAHs on thyroid development or function in human. Totally, PAHs decrease the circulating and tissue levels of thyroid hormones through at least three mechanisms: 1) PAHs may directly interfere with thyroid gland function and then change the structure of thyroid gland that lead to disruption of hormone synthesis [32]. 2) PAHs can target the metabolism of thyroid hormone. They may affect 5′-iodothyronine deiodinases (enzymes that convert T4 to T3 in target tissues). 3) PAHs may attach to thyroid hormone binding proteins in the blood stream [32]. These organic chemicals (PAHs) presented in the priority pollutant list of the United States Environmental Protection Agency (US-EPA 1998) because of their mutagenic, carcinogenic, and immunosuppressive properties [33].

Our findings showed a significant increase in PH frequencies in residents from Region 1, and multiple comparisons indicated that the PH odds were similar in districts that were closer to the CPC but different from the districts farther away, which were similar among themselves (Table 1).

The higher concentrations of NO_2_, CO, and VOCs, above the air quality standards, are inside of the CPC area (Table 2 and Table 3). In those places, studies were made regarding the frequency of PH (Table 1).

It is important to know the atmospheric lifetime of VOCs to gain an understanding of the distances they might travel in air. A higher atmospheric lifetime indicates that the VOCs can travel a greater distance in the atmosphere, possibly leading to impacts much farther away from the emission source [34].

In this study, the average concentrations of NO_2_, CO, PM_10_, SO_2_, and VOCs obtained from the simulated plumes in the six districts are larger in the three districts closer to the CPC (Table 2) and NO_2_, CO, and VOCs are the pollutants commonly related to the development of PH (Figure 2).

Because petroleum refining and petrochemical industries produce emissions at many stages of their operations—releasing pollutants such as volatile organic compounds, greenhouse gases, and particulate matter [34]—we believe that our findings are related with the CPC emissions, and these emissions can significantly lower the air quality and cause short-term and long-term health impacts for workers, and people living near the sites and in the same region.

Observations by health professionals in numerous parts of the world are increasingly bringing pollution, among the various factors thought to trigger CAT, into the spotlight of scientific interest. In an experimental study assessing thyroid function in Sprague-Dawley rats after exposure to Aroclor (PCBs group), distinct histopathological changes—such as hyperplasia of epithelia in follicles, reduction of colloid content, and lymphocytic infiltration into the perifollicular areas—were observed [35]. In parallel, the decreased free triiodothyronine (FT3) and free thyroxine (FT4) and the increased thyroid stimulating hormone (TSH) serum concentrations and antithyroperoxidase antibody (A-TPO) titers indicated that PCBs affect thyroid function [35].

Recently, a study in Brazil assessing the prevalence of Hashimoto’s thyroiditis (HT) and antithyroid antibodies (ATA) in residents of an area surrounding of a CPC reported higher incidence of both HT (9.3%) and ATA (17.6%) [36]. This study of the Epidemiological Research Center of the São Paulo State Department of Health in Brazil corroborated our findings of the increasing of CAT incidence over the years [21].

The concentration of iodine has been considered a modulating factor for thyroid autoimmunity. In iodine sufficient regions the major cause of PH is CAT [37] and iodine deficiency is the most common cause of PH worldwide [38]. In this study, iodine excess or deficiency were rejected as potential explanations of the PH odds because iodine sufficiency was demonstrated in a previous study in Region 1 [36,39,40].

Thus, in this study, atmospheric pollutants have been demonstrated to be the most likely cause of PH and shows that living in the surroundings of a CPC may be an important risk factor for PH for adults, children, and adolescents.

The adverse effects of air pollution are well established and documented. Several researchers in the field of public health, environmental health, international health—as well as international organizations and agencies—have done important contribution in the studying and publishing effects of environmental air pollution and human health is several scientific journals.

However, the effects of synthetic chemicals on thyroid function have received little attention, and there is much controversy over their potential clinical impact, because few studies have been conducted in humans.

## 6. Conclusions

Our data provide some evidence in favor of air pollution associated with the presence of a CPC being an important environmental factor contributing to the development of PH in the nearby population and, as the first study showing this association in Brazil, it should be continued to better understand the mechanism and find ways to compensate or remedy and avoid future population damages. 

## Figures and Tables

**Table 1:** Frequencies of residents (N), primary hypothyroidism (PH), odds of PH, and standard errors according to the multiple comparison procedure in adults, and children and adolescents (C/A) in Region 1 and Region 2.

Age	Sex	Districts	N	PH	Odds	Std. Error	Grouping
Adult	Female	Region 1	132	85	1.81	0.33	a
			151	56	0.59	0.10	b
			143	39	0.38	0.07	b
		Region 2	164	23	0.16	0.04	c
			193	27	0.16	0.03	c
			143	6	0.04	0.02	d
Adult	Male	Region 1	94	39	0.71	0.15	a
			89	24	0.37	0.09	a
			94	20	0.27	0.07	a
		Region 2	82	2	0.03	0.02	b
			72	0	0		b
			126	4	0.03	0.02	b
C/A	Female	Region 1	68	16	0.31	0.09	a
			51	10	0.24	0.09	a
			51	14	0.38	0.12	a
		Region 2	40	2	0.05	0.04	b
			42	1	0.02	0.02	b
			21	0	0	-	b
C/A	Male	Region 1	40	11	0.38	0.13	a
			43	8	0.23	0.09	a
			46	2	0.05	0.03	a
		Region 2	48	0	0		b
			27	0	0		b
			44	0	0		b

Total 2004 389.

**Table 2:** Average concentrations of NO_2_, CO, PM_10_, SO_2_, and VOCs obtained via the simulated plume along with the distance from the CPC

Location	Distance (Km)	NO_2_	CO	PM_10_	SO_2_	VOCs
		(µg/m^3^)	(µg/m^3^)	(µg/m^3^)	(µg/m^3^)	(µg/m^3^)
Region 1	0.5	13.16	3.95	0.93	1.36	477.4
	1	10.21	2.84	1.49	3.54	313.0
	2	4.32	1.21	0.83	2.03	69.3
Region 2	7.5	2.15	0.69	0.19	0.34	10.5
	8	1.82	0.59	0.16	0.33	9.2
	8.5	1.84	0.59	0.16	0.31	10.3

**Table 3:** Spearman correlation coefficients between the odds of PH and pollutants concentrations in adults, and children and adolescents (C/A)

Age	Sex	NO_2_	CO	PM_10_	SO_2_	VOCs
Adult	Female	0.94	0.94	0.89	0.83	0.94
	Male	0.94	0.94	0.89	0.66	0.94
C/A	Female	0.77	0.77	0.71	0.83	0.77
	Male	0.94	0.94	0.88	0.76	0.94
